# Magnetic field-induced magnetostructural transition and huge tensile superelasticity in an oligocrystalline Ni–Cu–Co–Mn–In microwire

**DOI:** 10.1107/S2052252519009102

**Published:** 2019-07-11

**Authors:** Zhen Chen, Daoyong Cong, Xiaoming Sun, Yin Zhang, Haile Yan, Shaohui Li, Runguang Li, Zhihua Nie, Yang Ren, Yandong Wang

**Affiliations:** a Beijing Advanced Innovation Center for Materials Genome Engineering, State Key Laboratory for Advanced Metals and Materials, University of Science and Technology Beijing, Beijing 100083, People’s Republic of China; b Key Laboratory for Anisotropy and Texture of Materials (Ministry of Education), Northeastern University, Shenyang 110819, People’s Republic of China; c School of Materials Science and Engineering, Beijing Institute of Technology, Beijing 100081, People’s Republic of China; d X-ray Science Division, Argonne National Laboratory, Argonne, IL 60439, USA

**Keywords:** superelasticity, microwires, magnetostructural coupling, martensitic transformations, shape-memory alloys, crystal structure

## Abstract

Meta-magnetic shape-memory alloys combine ferroelastic order with ferromagnetic order and exhibit attractive multifunctional properties, but they are extremely brittle, showing hardly any tensile deformability, which impedes their practical application. Here, for the first time, an Ni–Cu–Co–Mn–In microwire has been developed that simultaneously exhibits a magnetic field-induced first-order meta-magnetic phase transition and huge tensile superelasticity. A temperature-dependent *in situ* synchrotron high-energy X-ray diffraction investigation reveals that the martensite of this Ni_43.7_Cu_1.5_Co_5.1_Mn_36.7_In_13_ microwire shows a monoclinic six-layered modulated structure and the austenite shows a cubic structure. This microwire exhibits an oligocrystalline structure with bamboo grains, which remarkably reduces the strain incompatibility during deformation and martensitic transformation. As a result, huge tensile superelasticity with a recoverable strain of 13% is achieved in the microwire. This huge tensile superelasticity is in agreement with our theoretical calculations based on the crystal structure and lattice correspondence of austenite and martensite and the crystallographic orientation of the grains. Owing to the large magnetization difference between austenite and martensite, a pronounced magnetic field-induced magnetostructural transition is achieved in the microwire, which could give rise to a variety of magnetically driven functional properties. For example, a large magnetocaloric effect with an isothermal entropy change of 12.7 J kg^−1^ K^−1^ (under 5 T) is obtained. The realization of magnetic-field- and tensile-stress-induced structural transformations in the microwire may pave the way for exploiting the multifunctional properties under the coupling of magnetic field and stress for applications in miniature multifunctional devices.

## Abstract 2

An Ni–Cu–Co–Mn–In microwire that simultaneously exhibits a magnetic field-induced first-order magnetostructural transition (between the monoclinic six-layered modulated martensite and the cubic austenite) and huge tensile superelasticity has been developed. The huge tensile superelasticity is in agreement with theoretical calculations based on the crystal structure and lattice correspondence of austenite and martensite and the crystallographic orientation of the grains.

## 1. Introduction   

High-performance intelligent materials are important for the intelligent systems that are greatly required in our modern society. Shape-memory alloys (SMAs) are a unique class of intelligent materials which can recover their original shape during heating after being deformed in the low-temperature martensitic phase (Otsuka & Wayman, 1998 ▸). Thermoelastic martensitic transformation between the high-symmetry austenite and low-symmetry martensite is the basis of the shape-memory effect. Owing to the slow process of heating and cooling that controls the martensitic transformation, conventional SMAs exhibit a low-frequency response, which limits their application in many critical areas.

Magnetic SMAs that can be actuated by magnetic fields (with a high-frequency response) have drawn much attention during the past two decades (Ullakko *et al.*, 1996 ▸; Karaca *et al.*, 2009 ▸). The main actuation mechanisms of these alloys are: (i) magnetic field-induced martensitic variant reorientation (typical alloys with this actuation mechanism are the Ni–Mn–Ga alloys; Karaca *et al.*, 2006 ▸; Li *et al.*, 2012 ▸, 2014 ▸); and (ii) magnetic field-induced meta-magnetic phase transformation (the representative alloys will be mentioned later; Kainuma *et al.*, 2006 ▸; Murakami *et al.*, 2006 ▸). Alloys displaying a magnetic field-induced meta-magnetic phase transformation between austenite and martensite, due to the strong coupling between the crystallographic and magnetic structures, are called meta-magnetic SMAs (MMSMAs; Umetsu *et al.*, 2016 ▸). In particular, MMSMAs combine ferroelastic order and ferromagnetic order and they are typical multiferroic materials. As a result of the magnetic field-induced magnetostructural transition, these materials exhibit outstanding multifunctional properties such as the magnetic shape-memory effect (Kainuma *et al.*, 2006 ▸), magnetic superelasticity (Krenke *et al.*, 2007 ▸; Mañosa *et al.*, 2008 ▸), the magnetocaloric effect (Huang *et al.*, 2016 ▸; Liu *et al.*, 2009 ▸, 2012 ▸), magnetoresistance (Pathak *et al.*, 2010 ▸) and magnetothermal conductivity (Zhang *et al.*, 2007 ▸). Furthermore, the fact that the martensitic transformation in these materials can be induced by a change in magnetic field, stress or temperature offers a unique opportunity for optimizing the multifunctional properties under the coupling of multiple external fields.

Up to now, MMSMAs have mainly been discovered in the Ni–(Co)–Mn–*X* (*X* = In, Sn, Sb) and Fe–Mn–Ga alloy families. Unfortunately, polycrystalline MMSMAs are intrinsically brittle due to intergranular fracture arising from incompatibility at grain boundaries and triple junctions (Ueland *et al.*, 2012 ▸), which acts as a bottleneck for the practical application of MMSMAs. What is more, their high brittleness makes it rather difficult to harness the multifunctional properties under the simultaneous application of magnetic field and stress that is often encountered in real applications (Liu *et al.*, 2012 ▸; Karaca *et al.*, 2009 ▸). It is imperative to develop high-performance materials with both a magnetic field-induced first-order phase transition and extraordinary mechanical properties.

Producing an oligocrystalline structure in which the surface area is larger than the total grain boundary area and the triple junctions are reduced or even eliminated could diminish the incompatibility between different grains, allowing the deformation and martensitic transformation in SMAs to occur in a much less constrained environment (Ueland *et al.*, 2012 ▸). Consequently, this could effectively inhibit brittle intergranular fracture and enhance the mechanical properties (Chen *et al.*, 2009 ▸). The Taylor–Ulitovsky method, which involves the quenching and drawing technique (Chiriac & Óvári, 1996 ▸; Vázquez *et al.*, 2011 ▸), has been shown to be a feasible and cost-effective way of fabricating oligocrystalline-structured microwires of conventional SMAs (Ueland & Schuh, 2012 ▸). Several attempts have been made to prepare microwires of MMSMAs using the Taylor–Ulitovsky method (Qu *et al.*, 2017*a* ▸; Liu *et al.*, 2017 ▸; Li *et al.*, 2018 ▸; Vega *et al.*, 2012 ▸). However, a magnetic field-induced first-order meta-magnetic phase transition and large tensile superelasticity have never been simultaneously achieved.

Here in this work, we have successfully developed an oligocrystalline Ni–Cu–Co–Mn–In microwire exhibiting both a pronounced magnetic field-induced magnetostructural transition and huge tensile superelasticity with a recoverable strain of 13%. In this microwire, a small amount of Cu is added to improve the ductility, and the formation of an oligocrystalline structure effectively suppresses brittle intergranular fracture. The huge tensile superelasticity in the microwire is in sharp contrast with the awful tensile deformability of bulk MMSMAs. To our knowledge, this is the first report of a material that simultaneously exhibits a magnetic field-induced first-order meta-magnetic phase transition and excellent tensile superelasticity. The present microwire, showing both a reversible magnetic field-induced magnetostructural transition and tensile superelasticity, has enormous potential for applications in miniature multifunctional devices.

## 2. Experimental   

Button ingots with a composition of Ni_43.7_Cu_1.5_Co_5.1_Mn_36.7_In_13_ (at.%) were prepared by arc-melting of the pure Ni, Cu, Co and In elements and the master alloy Ni_40_Mn_60_ under an argon atmosphere. The Cu is added to enhance the ductility (Wang *et al.*, 2010 ▸) and to tune the martensitic transformation temperature and Curie temperature (Das *et al.*, 2011 ▸). In order to ensure homogeneity, the ingots were melted four times. Then a part of the button ingot was remelted and cast into a copper mould to prepare a cylindrical rod with a diameter of 5 mm, which was subsequently used for microwire preparation. Glass-coated microwires with diameters of 100−200 µm were fabricated using the Taylor–Ulitovsky method (Chiriac & Óvári, 1996 ▸; Vázquez *et al.*, 2011 ▸). The glass sheath on the microwire was removed by grinding on fine sand paper. The microwires were tested in the as-drawn state. To determine the phase transition temperature and transition entropy change of the microwires, differential scanning calorimetry (DSC) measurements were conducted, with cooling and heating rates of 10 K min^−1^.

The crystal structures of austenite and martensite and the evolution of crystal structure during cooling and heating in the temperature range 300−110 K were studied by employing the *in situ* synchrotron high-energy X-ray diffraction (HEXRD) technique. The *in situ* HEXRD experiments were conducted on the 11-ID-C beamline at the Advanced Photon Source, Argonne National Laboratory, USA, employing a monochromatic X-ray beam with a wavelength of 0.1173 Å. The diffraction patterns were collected with a 2D image-plate detector. The relative orientation of the incident X-ray beam with respect to the wire samples is shown in Fig. S1 (supporting information). The surface morphology and fractography of the microwire were studied using a scanning electron microscope (SEM). The crystallographic orientation of the microwire was measured by the electron backscatter diffraction (EBSD) technique in the SEM. More detailed information on the EBSD measurements for crystallographic analysis in magnetic SMAs can be found in the literature (Zhang *et al.*, 2016*a* ▸, 2017*a* ▸; Lin *et al.*, 2016 ▸; Yan *et al.*, 2016 ▸).

The tensile properties of the microwire were measured at different temperatures using a mechanical testing machine (Instron 5966, with a 100 N load cell) equipped with a temperature chamber. The gauge length of the microwire is about 8 mm. The tensile tests were performed by displacement-controlled loading at a low strain rate of 5 × 10^−4^ s^−1^. The magnetization of the microwire as a function of temperature and magnetic field was measured with a physical property measurement system (PPMS, Quantum Design). The magnetization versus temperature [*M*(*T*)] curves were measured under 0.05 and 5 T with heating and cooling rates of 5 K min^−1^. The magnetization versus field [*M*(*H*)] curves were measured at different temperatures during two cycles of field changes of 0→5 T→0. The standard loop process was used: before the *M*(*H*) measurement at each temperature, the microwire was first cooled to 130 K and then heated to the measurement temperature.

## 3. Results and discussion   

### 3.1. Crystal structure and temperature-induced phase transition   

**Figure 1:**
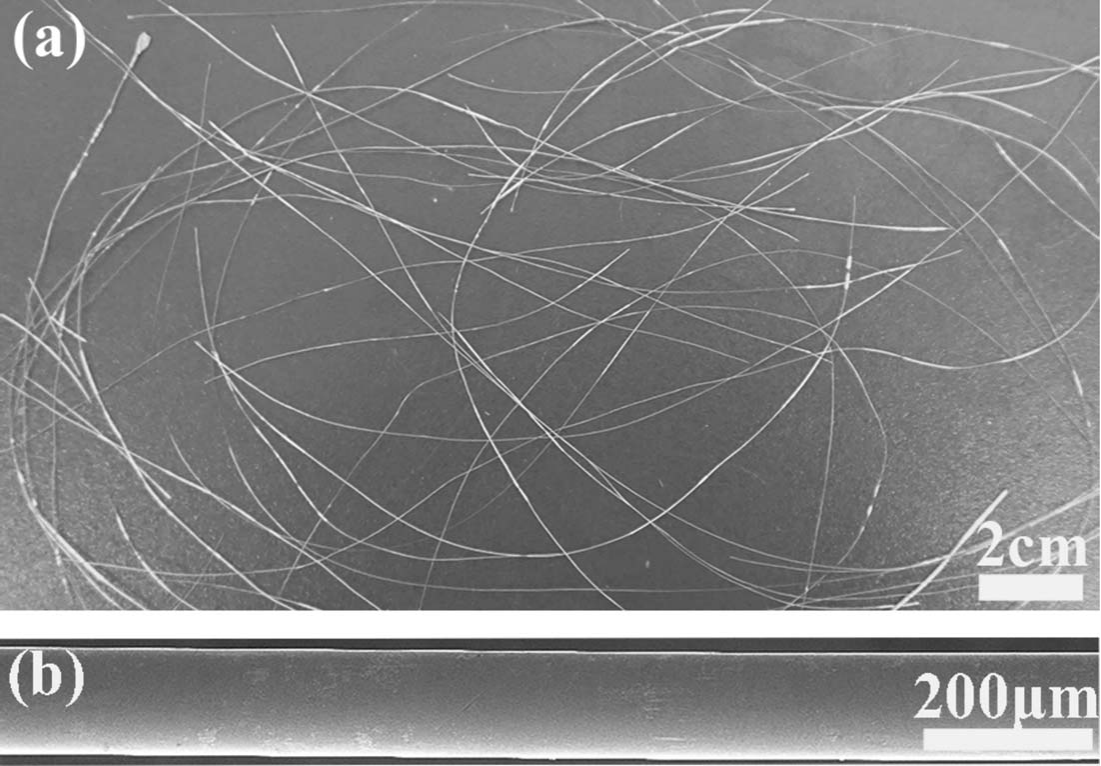
(*a*) A photograph of the Ni–Cu–Co–Mn–In microwires. (*b*) An SEM image showing the surface morphology of an Ni–Cu–Co–Mn–In microwire.

Fig. 1 ▸(*a*) shows a photograph of the Ni–Cu–Co–Mn–In microwires, demonstrating that continuous microwires with lengths of tens of centimetres can be successfully fabricated with the Taylor–Ulitovsky method. The surface morphology of a typical microwire is displayed in Fig. 1 ▸(*b*), showing that the microwire has a uniform diameter and a smooth surface. This is beneficial for reducing the dissipation energy during a superelastic cycle (Ueland & Schuh, 2014 ▸). The EBSD measurement reveals that the microwires exhibit an oligocrystalline structure with bamboo grains, as demonstrated in Section 3.2. This kind of oligocrystalline structure is beneficial for diminishing the incompatibility between adjacent grains and inhibiting brittle intergranular fracture.

**Figure 2:**
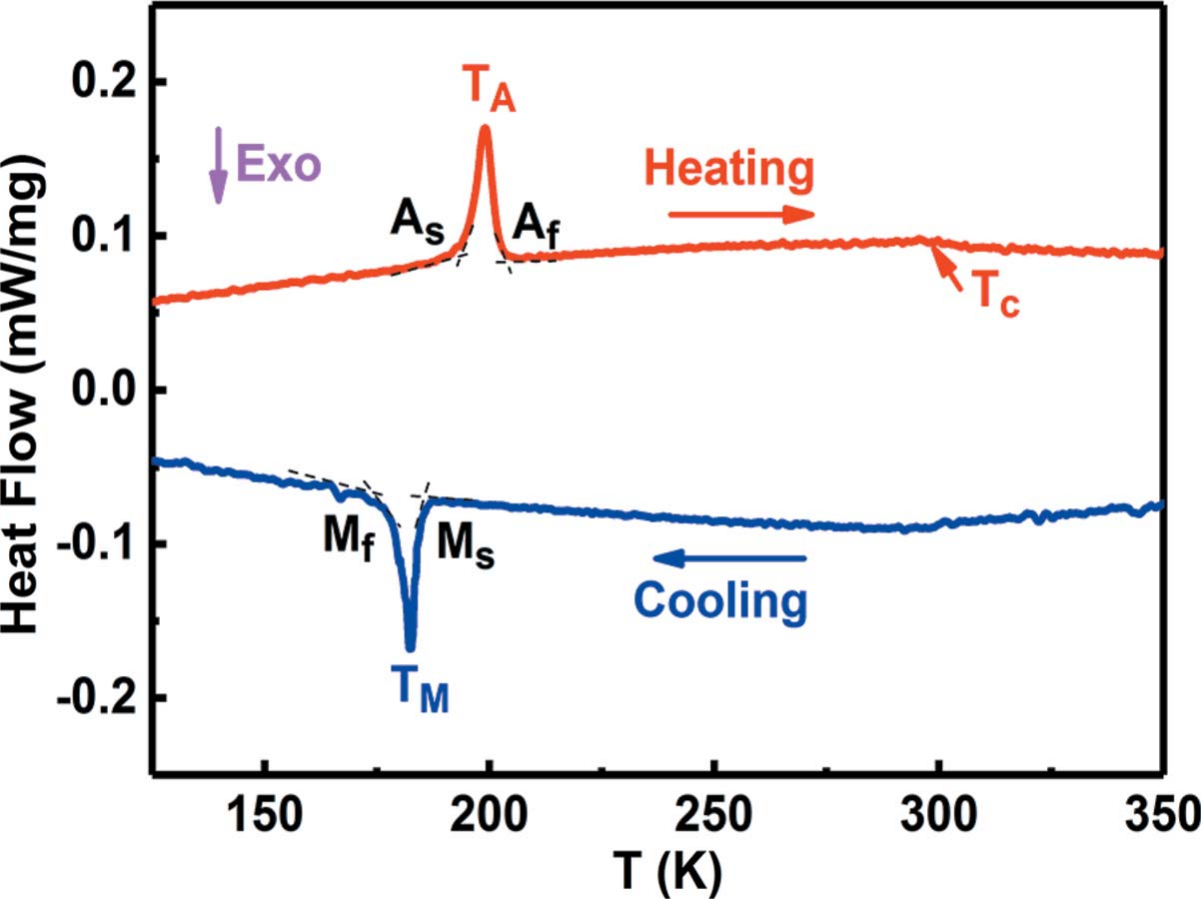
Heating and cooling DSC curves for the Ni_43.7_Cu_1.5_Co_5.1_Mn_36.7_In_13_ microwire. The phase transformation temperatures are determined as illustrated in the figure. The Curie temperature of austenite *T*_c_ is denoted by an arrow.

The DSC curves of the Ni_43.7_Cu_1.5_Co_5.1_Mn_36.7_In_13_ microwire are shown in Fig. 2 ▸. The pronounced exothermic and endothermic peaks correspond to the first-order martensitic and reverse transformations, respectively. The martensitic and reverse transformation start, finish and peak temperatures *M*_s_, *M*_f_, *T*_M_, *A*_s_, *A*_f_ and *T*_A_ are 185.3, 175.1, 182.3, 193.3, 202.5 and 199.0 K, respectively. The thermal hysteresis, estimated as Δ*T*_hys_ = (*A*_s_ + *A*_f_ − *M*_s_ − *M*_f_)/2, is 17.7 K. The Curie temperature of austenite *T*_c_, which is indicated by a red arrow in Fig. 2 ▸, is identified to be 300.8 K. These temperatures are also listed in Table 1 ▸. The temperature difference between *T*_c_ and *T*_A_ is very large (101.8 K); this is beneficial for realizing a magnetic field-induced phase transition in the microwire since a larger *T*_c_ − *T*_A_ leads to a higher sensitivity of the transition temperature to magnetic field (Gottschall *et al.*, 2016 ▸; Recarte *et al.*, 2012 ▸). The entropy change for the reverse transformation, Δ*S*_tr_, is estimated from the endothermic peak to be 12.9 J kg^−1^ K^−1^.

**Figure 3:**
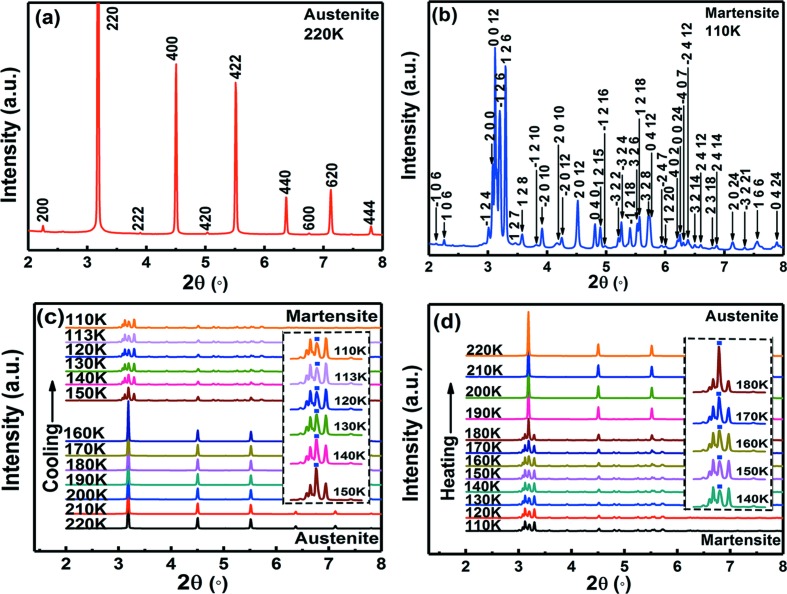
(*a*), (*b*) 1D HEXRD patterns of the Ni_43.7_Cu_1.5_Co_5.1_Mn_36.7_In_13_ microwire collected at (*a*) 220 K and (*b*) 110 K during cooling, and the indexing of the patterns. The 1D patterns are obtained by integrating the 2D patterns along all azimuth angles. (*c*), (*d*) Evolution of the HEXRD patterns during (*c*) cooling from 220 to 110 K and (*d*) heating from 110 to 220 K. The insets in (*c*) and (*d*) show magnified views of the patterns in the 2θ range between 2.9° and 3.7°. The blue squares denote the {220} peak of austenite and the {−126} peak of martensite.

In order to determine the crystal structures of austenite and martensite and to gain deep insights into the phase transition behaviour from the structural point of view, *in situ* HEXRD experiments were conducted to trace the crystal structure evolution during cooling and heating. The HEXRD technique has the advantages of high penetration, low absorption and high resolution, making it an ideal tool for detecting structural evolution in microwires. The 1D HEXRD patterns of the Ni_43.7_Cu_1.5_Co_5.1_Mn_36.7_In_13_ microwire, collected at 220 and 110 K during cooling, are displayed in Figs. 3 ▸(*a*) and 3 ▸(*b*), respectively. At 220 K, strong {220}, {400} and {422} diffraction peaks are observed, but the {111} and {311} superlattice diffraction peaks are not visible. This diffraction pattern [Fig. 3 ▸(*a*)] can be indexed according to the B2 structure (space group 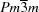, No. 221) of austenite with lattice parameter *a* = 2.9865 Å. In this sense, the as-drawn microwires may show a higher disorder than the annealed bulk alloys that usually possess an L2_1_ Heusler austenitic structure (Liu *et al.*, 2015 ▸). Nevertheless, considering that the intensities of the {111} and {311} superlattice diffraction peaks are usually weak and the relative orientation of the incident X-ray beam with respect to the sample (Fig. S1) may have a significant influence on the intensities of these peaks because the microwire used for the HEXRD experiment is in the oligocrystalline form (different from randomly oriented powders), at present the L2_1_ Heusler structure of austenite cannot be excluded; the diffraction pattern in Fig. 3 ▸(*a*) can also be indexed according to the L2_1_ Heusler structure (space group 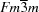, No. 225) with lattice parameter *a* = 5.9730 Å. The HEXRD pattern at 110 K can be well indexed according to the monoclinic six-layered modulated (6M) martensitic structure (space group *P*2/*m*, No. 10). The indexing of the pattern is shown in Fig. 3 ▸(*b*). The lattice parameters are determined as *a*_6M_ = 4.3725 Å, *b*_6M_ = 5.5950 Å, *c*_6M_ = 25.8861 Å and β = 93.5660°.

To reveal the structure evolution during the phase transition, HEXRD patterns were collected while the microwire was cooled down from 300 to 110 K and then heated back up to 300 K. The evolution of the HEXRD patterns during cooling from 220 to 110 K and heating from 110 to 220 K is demonstrated in Figs. 3 ▸(*c*) and 3 ▸(*d*), respectively. As seen from Fig. 3 ▸(*c*), upon cooling from 160 to 150 K, the vast majority of austenite transforms into martensite within this temperature interval of 10 K. In the temperature region within which austenite and martensite coexist, the {220} peak of austenite [see Fig. 3 ▸(*a*)] and the {−126} peak of martensite [see Fig. 3 ▸(*b*)] overlap, as marked by the blue squares in the insets of Figs. 3 ▸(*c*) and 3 ▸(*d*). Upon further cooling below 150 K, the intensity of this overlapped peak decreases while the intensities of the other martensitic peaks increase, as seen from the inset of Fig. 3 ▸(*c*). This indicates that the untransformed austenite keeps continuously and gradually transforming into martensite. Comparing the HEXRD patterns at 113 and 110 K, one can see that the pattern does not change any more upon cooling below 113 K, which implies that the phase transition is complete at 113 K. In the low-temperature region of 150−113 K, the coexistence of martensite and austenite may result from the extremely low mobility of the habit plane between austenite and martensite at such low temperatures (Ito *et al.*, 2008 ▸). As shown in Fig. 3 ▸(*d*), upon heating the reverse transition occurs in a similar way. The martensite transforms continuously and gradually into austenite in the temperature region of 140−180 K and the majority of martensite transforms completely into austenite between 180 and 190 K.

It should be mentioned that the phase transition temperatures in the HEXRD experiment are somewhat different from those determined from the DSC measurement. This is explained as follows. Due to the complexity of the experimental setup and the small size of the microwire, the thermo­couple was located a few centimetres away from the microwire. This, as well as the high ramp rate (*ca* 10 K min^−1^), leads to the fact that the temperature monitored by the thermocouple may not reflect the real temperature of the microwire. In addition, it should be noted that the continuous and gradual transition from austenite to martensite during cooling (after the vast majority of the transition which is detectable by DSC measurement) only releases a small amount of heat which is insufficient to form a visible peak on the DSC curve and therefore cannot be detected by DSC measurement.

The geometric compatibility between austenite and martensite, which shows an intimate relationship with the thermal hysteresis, was evaluated on the basis of the geometric nonlinear theory of martensite using the crystal symmetry and lattice parameters of austenite and martensite as determined above from the HEXRD experiment. The middle eigenvalue λ_2_ of the transformation stretch matrix **U**, which is a quantitative measure of the geometric compatibility, can be determined with the algorithms given in the literature (Song *et al.*, 2013 ▸; Zarnetta *et al.*, 2010 ▸; Hane & Shield, 1999 ▸). The λ_2_ of the present Ni_43.7_Cu_1.5_Co_5.1_Mn_36.7_In_13_ microwire is determined to be 0.9952 (*i.e.* |1 − λ_2_| = 0.0048, the same for both L2_1_ and B2 structures of austenite), which is close to unity. The norms *X*_I_ and *X*_II_ are also computed according to the algorithms reported in the literature (Chen *et al.*, 2013 ▸) and they are 1.0075 and 1.0002, respectively. The unit-cell volume change upon the transformation from austenite to 6M martensite during cooling is determined to be −1.13%.

### 3.2. Tensile superelasticity and its interpretation based on crystallography   

**Figure 4:**
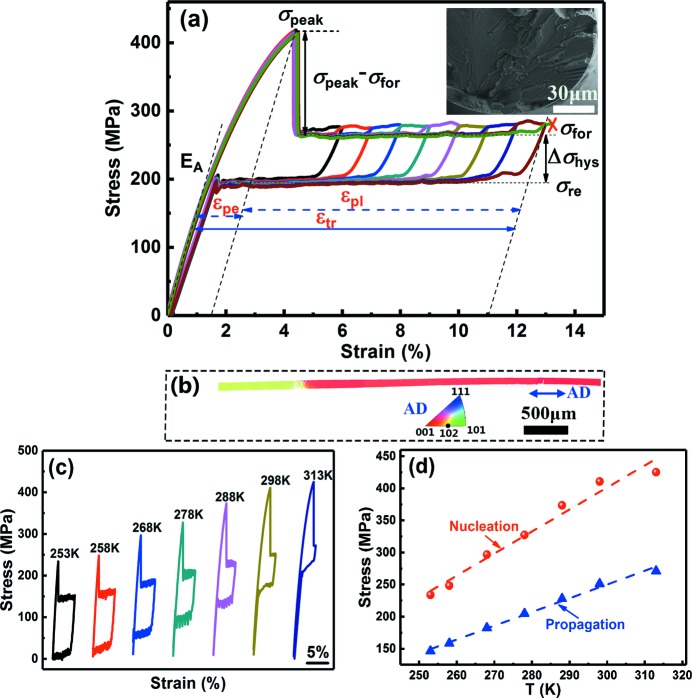
(*a*) Tensile stress–strain curves of the Ni_43.7_Cu_1.5_Co_5.1_Mn_36.7_In_13_ microwire, measured up to different strain levels at room temperature. The symbol (×) represents the point of fracture. The upper right inset shows a fractograph after the tensile test. (*b*) An EBSD orientation map of the tested microwire. This map is presented in inverse pole-figure mode; the legend (parallel to AD) is also displayed in the figure. AD denotes the axial direction of the microwire. (*c*) Tensile stress–strain curves measured at different temperatures from 253 to 313 K. (*d*) The temperature dependence of the nucleation stress and propagation stress.

To examine the tensile superelasticity as a result of stress-induced martensitic transformation, the tensile stress–strain curves during loading and unloading at different temperatures above *A*_f_ were measured. Fig. 4 ▸(*a*) shows the room-temperature stress–strain curves for an Ni_43.7_Cu_1.5_Co_5.1_Mn_36.7_In_13_ microwire with a diameter of 144 µm. The superelastic cycles were measured sequentially with increasing maximum strain level until failure. As can be seen, the microwire displays excellent tensile superelasticity with almost no residual strain for all the superelastic cycles. Strikingly, the tensile recoverable strain is as high as 13%. This is in sharp contrast with bulk MMSMAs that exhibit hardly any tensile deformability.

As seen from Fig. 4 ▸(*a*), upon loading the austenite first deforms elastically with strain up to 1.1% (the elastic modulus of austenite *E*_A_ is indicated in the figure). Then the stress–strain path deviates from linearity. When the strain reaches 4.1%, the stress drops dramatically from 416.1 to 264.6 MPa. This is because the deformation mechanism changes from martensite nucleation to martensite propagation via phase-front motion. The peak stress σ_peak_ [as indicated in Fig. 4 ▸(*a*)] and the large peak strain ∊_pe_ [as indicated in Fig. 4 ▸(*a*)] suggest martensite nucleation and possibly some growth (Monroe *et al.*, 2010 ▸) before the stress drop. The peak stress σ_peak_ corresponds to the nucleation stress (Shaw, 2000 ▸), which is the critical stress required for martensite nucleation. After the sudden stress drop, a stress plateau appears, which corresponds to the propagation stress of forward martensitic transformation (Shaw, 2000 ▸), indicated as σ_for_ in Fig. 4 ▸(*a*). The stress difference between nucleation stress σ_peak_ and propagation stress σ_for_, σ_peak_ − σ_for_, which represents the height of the nucleation peak, is 151.5 MPa.

As seen from Fig. 4 ▸(*a*), during the cyclic measurements the propagation stress of the cycle with a higher maximum strain is generally lower than that of the previous cycle before meeting the point where the previous cycle was interrupted, but beyond that point the propagation stress increases by about 15 MPa to match the propagation stress of the previous cycle. This phenomenon was also observed in Cu–Zn–Al and Ni–Ti shape-memory wires (Ueland & Schuh, 2012 ▸; Yawny *et al.*, 2005 ▸). It can be explained as follows. The dislocations introduced during the previous cycle create internal stress fields that favour the growth of oriented martensitic variants, which results in lower stresses during subsequent reloading before meeting the interruption point (Yawny *et al.*, 2005 ▸; Simon *et al.*, 2010 ▸). However, once the applied strain exceeds the maximum strain of the previous cycle, additional dislocations are introduced during further transformation, and therefore the propagation stress increases to match the level of the previous cycle (Ueland & Schuh, 2012 ▸).

During unloading, the stress first decreases linearly and then remains almost constant, forming a lower stress plateau. The plateau stress of reverse transformation from martensite to austenite, shown as σ_re_ in Fig. 4 ▸(*a*), is 194.7 MPa.

The stress hysteresis Δσ_hys_ is determined from the difference between σ_for_ and σ_re_. As indicated in Fig. 4 ▸(*a*), Δσ_hys_ is almost the same for all the superelastic cycles and it remains at a value of 69.9 MPa. The fact that the size of Δσ_hys_ hardly depends on the maximum applied strain indicates the microstructural stability of the microwire (Panchenko *et al.*, 2010 ▸).

When we attempted to measure the stress–strain curve with a maximum strain of 14%, the sample failed in the plateau region (at a strain of 13.2%), and thus the phase transition induced by stress is not completed. Therefore, the maximum plateau strain may be larger than the strain ∊_pl_ indicated in Fig. 4 ▸(*a*). In this sense, the maximum transformation strain (∊_tr_) in the microwire is larger than the sum of ∊_pe_ and ∊_pl_ [see Fig. 4 ▸(*a*) for the determination of ∊_pe_ and ∊_pl_].

A fractograph of the tested Ni_43.7_Cu_1.5_Co_5.1_Mn_36.7_In_13_ microwire is shown in the inset of Fig. 4 ▸(*a*). River-like patterns can be clearly seen, indicating that the fracture mode is transgranular fracture (Wang *et al.*, 2006 ▸). Small cleavage planes and cleavage steps, as well as tear ridges, can also be observed. The transgranular fracture in the microwire is in contrast to the intergranular fracture with rock candy patterns observed in polycrystalline bulk Ni–Mn–In alloys (Feng *et al.*, 2009 ▸). This confirms that intergranular fracture is indeed inhibited in the microwire.

It should be mentioned that the microwire fractured at a position close to the grip. The EBSD measurement was performed on the longer part of the fractured microwire, to examine the crystallographic orientation. The EBSD orientation map, presented in inverse pole-figure mode with respect to the axial direction (AD) of the microwire, is shown in Fig. 4 ▸(*b*). The right-hand side of the microwire is close to the fractured surface. Due to mechanical grinding and electrolytic polishing, the diameter of the microwire for the EBSD measurement becomes less than 144 µm (the initial diameter). Clearly, only two austenite grains can be observed from the EBSD orientation map [Fig. 4 ▸(*b*)]. Each grain spans the entire wire cross section and the grain boundary is oriented nearly perpendicular to the wire axis. This clearly demonstrates that the microwire exhibits an oligocrystalline structure with bamboo grains (Ueland *et al.*, 2012 ▸). The right-hand grain is larger than the left-hand one. The 〈001〉 direction of the right-hand grain and the 〈012〉 direction of the left-hand one are parallel to the wire axis. Both these directions are favourable for attaining a large transformation strain (as discussed later). Overall, the above oligocrystalline structure with bamboo grains is important for achieving the huge tensile superelasticity with a recoverable strain of 13% as mentioned above.

Stress–strain curves at different temperatures ranging from 253 to 313 K are shown in Fig. 4 ▸(*c*). Clearly, this Ni_43.7_Cu_1.5_Co_5.1_Mn_36.7_In_13_ microwire exhibits almost perfect tensile superelasticity with a recoverable strain of 5.5% at each temperature in this temperature interval of 60 K. Fig. 4 ▸(*d*) shows the temperature dependence of the nucleation stress and propagation stress of the stress-induced martensitic transformation. Clearly, both the nucleation stress and propagation stress increase linearly with increasing temperature, with rates of 3.43 and 2.14 MPa K^−1^, respectively. Extrapolation of the linear fit lines of nucleation stress versus temperature and propagation stress versus temperature to zero stress yields 184.8 and 184.5 K, respectively, both in good agreement with the value of *M*_s_ determined from the DSC measurement. As demonstrated above, we achieved excellent tensile superelasticity in the microwire in the temperature range between 253 and 313 K. This is impossible in bulk MMSMAs that can hardly be deformed in tension.

Huge tensile superelasticity with a recoverable strain of 13% was achieved in the Ni_43.7_Cu_1.5_Co_5.1_Mn_36.7_In_13_ microwire. As mentioned before, the maximum transformation strain (∊_tr_) in the microwire is larger than the sum of ∊_pe_ and ∊_pl_ shown in Fig. 4 ▸(*a*), which is 11%. EBSD measurement reveals that the microwire consists of two grains, with the 〈001〉 direction of the larger grain and the 〈012〉 direction of the smaller one parallel to the wire axis. In the following, we will interpret this tensile superelasticity by theoretical calculations based on the energy-minimization theory, which requires the crystal structure information of austenite and martensite and the lattice correspondence between the unit cells of these two phases as input parameters.

According to the energy-minimization theory (James & Hane, 2000 ▸), the strain associated with the formation of the most favourable correspondence variant pairs (CVP) is termed the CVP transformation strain (∊_CVP_). The detwinning strain (∊_det_) arises from the growth of one variant at the expense of the other within the CVP (Karaca *et al.*, 2009 ▸) when further loading is applied to the twinned martensite (Dadda *et al.*, 2008 ▸). The maximum theoretical transformation strain is the sum of ∊_CVP_ and ∊_det_. The detailed procedure for calculating ∊_CVP_ and the maximum theoretical transformation strain can be found in the work of James & Hane (2000 ▸) and Sehitoglu *et al.* (2000 ▸).

Since the thermally induced martensite shows a monoclinic six-layered modulated (6M) structure according to the HEXRD experiments, we first assume that the stress-induced martensite also shows the 6M structure. According to Karaca *et al.* (2009 ▸), for the transformation from cubic austenite to 6M martensite, the transformation strain along the 〈001〉 direction of austenite has the largest value. With the crystal structure information of austenite and 6M martensite determined from the HEXRD experiments (Section 3.1), we calculated ∊_CVP_ and the maximum theoretical transformation strain under tension along the 〈001〉 and 〈012〉 directions of austenite using the energy-minimization theory. When the loading is applied along 〈001〉, the twinning plane normal, the twinning shear direction, the habit plane normal and the transformation shear direction are determined to be *n* = {0.7781, 0.0000, −0.6281}, *a* = 〈0.0789, 0.0000, 0.1044〉, *m* = {0.0698, 0.7199, −0.6906} and *b* = 〈0.0078, −0.0902, −0.0768〉, respectively. ∊_CVP_ and the maximum theoretical transformation strain along 〈001〉 are 5.64% and 6.18%, respectively. When the loading is applied along 〈012〉, the twinning plane normal, the twinning shear direction, the habit plane normal and the transformation shear direction are determined to be *n* = {0.0000, 0.7071, −0.7072}, *a* = 〈0.0000, 0.0871, 0.0926〉, *m* = {−0.7198, 0.0910, 0.6882} and *b* = 〈0.0902, 0.0101, 0.0766〉, respectively. ∊_CVP_ and the maximum theoretical transformation strain along 〈012〉 are 5.09% and 5.42%, respectively. It should be noted that calculations using the L2_1_ and B2 structures of austenite yield the same results. Clearly, the maximum theoretical transformation strains along 〈001〉 and 〈012〉 are both much smaller than the experimentally observed transformation strain (11%) in the Ni_43.7_Cu_1.5_Co_5.1_Mn_36.7_In_13_ microwire. This indicates that our assumption that the stress-induced martensite shows a 6M structure is not reasonable. Therefore, the martensite induced by stress may show a different structure from that induced by temperature.

It was reported in Ni–Fe–Ga SMAs that the austenite transforms into five-layered modulated (5M) and seven-layered modulated (7M) martensites upon cooling (Oikawa *et al.*, 2002 ▸), and the 7M martensite transforms into the tetragonal non-modulated martensite during tension (Sutou *et al.*, 2004 ▸). It was also reported in Ni–Mn–Ga SMAs (Ge *et al.*, 2015 ▸; Pagounis *et al.*, 2015 ▸; Huang *et al.*, 2015 ▸) that the 7M martensite transforms into non-modulated martensite under compression. Furthermore, it was found that in an Ni_54_Ga_27_Fe_19_ single crystal the non-modulated martensite could be induced directly from austenite at a temperature much higher than *A*_f_ (Sutou *et al.*, 2004 ▸) and the corresponding stress–strain curve shows the characteristic feature that the stress decreases drastically after the stress peak and then a stress plateau appears, which is very similar to what is observed in the present Ni_43.7_Cu_1.5_Co_5.1_Mn_36.7_In_13_ microwire [Fig. 4 ▸(*a*)]. Therefore, it is quite probable that the non-modulated martensite is induced by tensile stress in the present microwire.

The lattice parameters of the tetragonal non-modulated (NM) martensite can be estimated from the monoclinic 6M martensitic structure using the method proposed by Kainuma *et al.* (1996 ▸) and Sutou *et al.* (2004 ▸), and the results are 
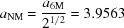
and 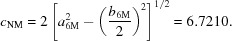
Based on the energy-minimization theory, the twinning plane normal, the twinning shear direction, the habit plane normal and the transformation shear direction were calculated, using the crystal structure information of austenite and NM martensite, to be *n* = {0, −0.7071, 0.7071}, *a* = 〈0, 0.2886, 0.2402〉, *m* = {−0.7243, 0.1023, 0.6818} and *b* = 〈0.0895, 0.0112, 0.0749〉, respectively, for both cases when the loading is applied along 〈001〉 and 〈012〉. ∊_CVP_ and the maximum theoretical transformation strain along 〈001〉 of austenite were calculated to be 5.43% and 13.31%, respectively. The value of ∊_det_ as large as 7.88% is comparable with that reported by Hamilton *et al.* (2007 ▸). The calculated ∊_CVP_ and maximum theoretical transformation strain along 〈012〉 are 5.02% and 9.42%, respectively. The above maximum theoretical transformation strains associated with the transformation from austenite to NM martensite are consistent with the experimentally observed transformation strain in the Ni_43.7_Cu_1.5_Co_5.1_Mn_36.7_In_13_ microwire.

As is well known, bulk MMSMAs exhibit hardly any tensile superelasticity due to their poor tensile deformability (Villa *et al.*, 2015 ▸). Even under compression, the recoverable superelastic strain in bulk MMSMAs is very limited, especially in polycrystalline bulk MMSMAs with random grain orientations. In contrast, the present Ni_43.7_Cu_1.5_Co_5.1_Mn_36.7_In_13_ microwire shows huge tensile superelasticity with a recoverable strain of as high as 13%. In addition to the favourable orientations for attaining a large transformation strain as mentioned above, the specific microstructure of the microwire, which is different from that of bulk polycrystals, plays a crucial role. There are no triple junctions and there is only a single grain boundary [Fig. 4 ▸(*b*)] in the oligocrystalline structure of the microwire. The grains are mostly surrounded by unconfined free surfaces, and strain accumulation during deformation and transformation can be easily relieved at the surfaces. In this way, the deformation and transformation strain incompatibility and the stress concentration (Liu *et al.*, 2014 ▸) are markedly reduced, allowing the deformation and martensitic transformation to occur in a much less constrained environment. Therefore, the present microwire can be easily deformed in tension and shows huge tensile superelasticity.

### 3.3. Magnetic field-induced magnetostructural transition and magnetically driven properties   

**Figure 5:**
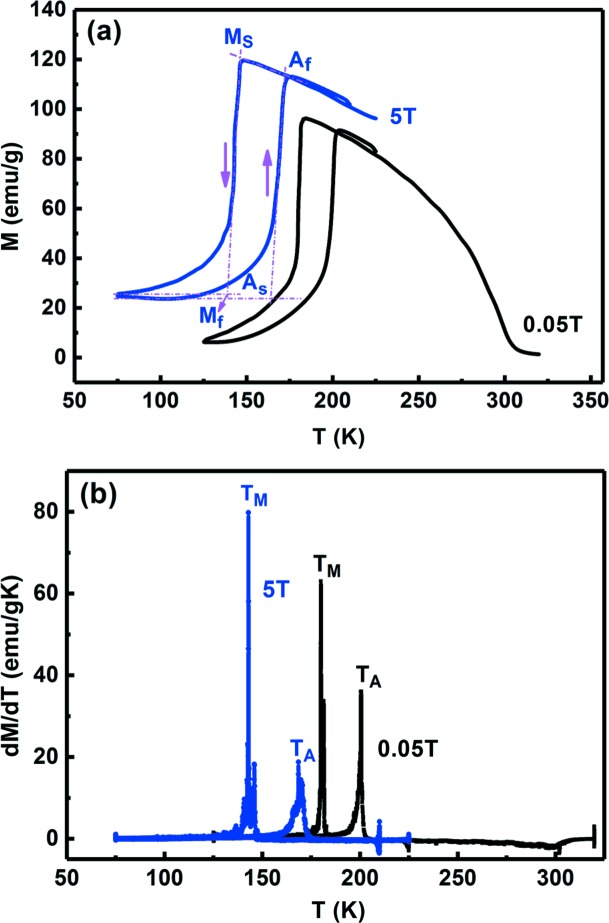
(*a*) *M*(*T*) curves measured under magnetic fields of 0.05 and 5 T for the Ni_43.7_Cu_1.5_Co_5.1_Mn_36.7_In_13_ microwire. The determination of phase transition temperatures is illustrated in the figure. (*b*) The temperature dependence of d*M*/d*T* derived from the *M*(*T*) curves in panel (*a*).

The *M*(*T*) curves under 0.05 and 5 T and the *M*(*H*) curves at different temperatures between 130 and 180 K for the Ni_43.7_Cu_1.5_Co_5.1_Mn_36.7_In_13_ microwire are shown in Figs. 5 ▸(*a*) and 6 ▸(*a*), respectively. The high-temperature austenite is ferromagnetic and the low-temperature martensite is weakly magnetic. There is a large magnetization difference Δ*M* of 90.3 emu g^−1^ across the phase transition, as determined from the *M*(*T*) curve under 5 T in Fig. 5 ▸(*a*). This indicates that a magnetic field-induced magnetostructural transition could be expected in this microwire.

As indicated from the *M*(*T*) curve under 0.05 T shown in Fig. 5 ▸(*a*), during cooling the martensitic transformation occurs intensively (with the vast majority of austenite transforming into martensite) between 185 and 175 K, and then the untransformed austenite transforms continuously and gradually into martensite upon further cooling. This is in qualitative agreement with the *in situ* HEXRD results as demonstrated in Section 3.1, taking into account the discrepancy in temperature measurement. The phase transition temperatures (*M*_s_, *M*_f_, *A*_s_ and *A*_f_) under 0.05 and 5 T, determined using the tangent intersection method from the *M*(*T*) curves in Fig. 5 ▸(*a*), are listed in Table 1 ▸. The temperature dependence of d*M*/d*T* derived from the *M*(*T*) curves [Fig. 5 ▸(*a*)] is shown in Fig. 5 ▸(*b*). The temperatures at which the maxima on the d*M*/d*T* versus *T* curves appear during cooling and heating correspond to the martensitic transformation peak temperature *T*_M_ and the reverse transformation peak temperature *T*_A_, respectively. The values of *T*_M_ and *T*_A_ under 0.05 and 5 T are also included in Table 1 ▸. Clearly, all the phase transition temperatures decrease under the magnetic field of 5 T, which is because the applied magnetic field stabilizes the austenite phase with a higher magnetization. Specifically, *T*_A_ decreases by 31.8 K upon application of a magnetic field of 5 T, with the field dependence of the transition temperature Δ*T*_A_/μ_0_Δ*H* being about −6.4 K T^−1^.

According to the Clausius–Clapeyron relation, the dependence of the reverse transformation temperature change (Δ*T*_A_) on the magnetic field change (μ_0_Δ*H*) satisfies the following relation (Kainuma *et al.*, 2006 ▸; Kustov *et al.*, 2009 ▸; Cong *et al.*, 2012 ▸): 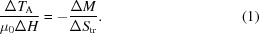
With the Δ*S*_tr_ value determined from the DSC measurement (12.9 J kg^−1^ K^−1^) and the Δ*M* value determined from the *M*(*T*) curve under 5 T (90.3 emu g^−1^), Δ*M*/Δ*S*_tr_ is computed to be 7.0 K T^−1^, which is in general agreement with the Δ*T*_A_/μ_0_Δ*H* value mentioned above. The fact that the phase transition temperatures can be significantly decreased by a magnetic field suggests that applying a magnetic field at a temperature close to the reverse transformation temperature could induce a first-order magnetostructural transition from the six-layered modulated (6M) martensite to the cubic austenite. The high value of Δ*T*_A_/μ_0_Δ*H* facilitates the achievement of a magnetic field-induced magnetostructural transition under a lower field.

**Figure 6:**
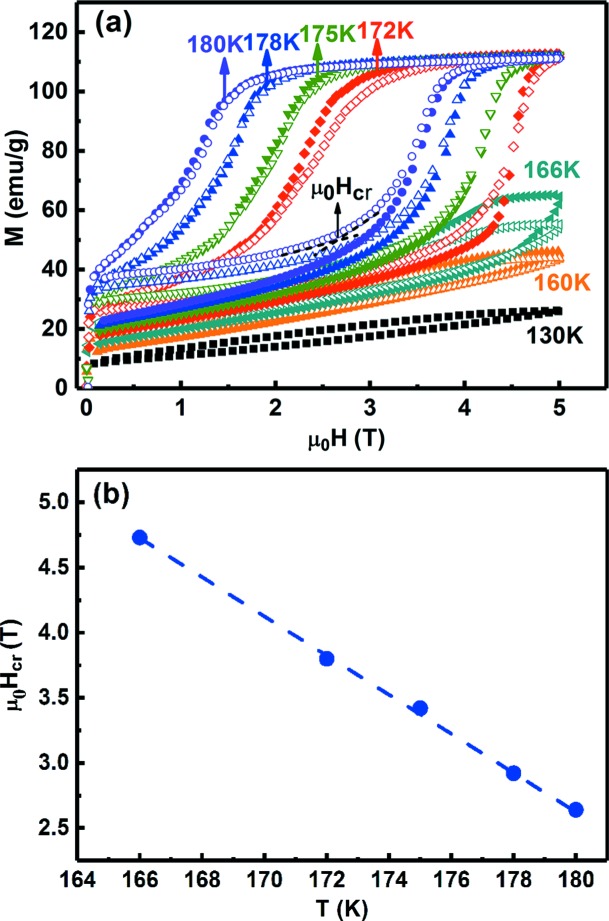
(*a*) *M*(*H*) curves measured during the first (open symbols) and second (solid symbols) cycles of increasing and decreasing field at different temperatures for the Ni_43.7_Cu_1.5_Co_5.1_Mn_36.7_In_13_ microwire. The determination of the critical field (μ_0_ *H*_cr_) for the magnetic field-induced phase transition is illustrated in the figure. (*b*) The temperature dependence of the critical field (μ_0_ *H*_cr_) extracted from the second cycle of *M*(*H*) curves in panel (*a*). The dashed line is the linear fit line of the data (shown as symbols).

In order to verify the magnetic field-induced phase transition in the Ni_43.7_Cu_1.5_Co_5.1_Mn_36.7_In_13_ microwire and its reversibility, *M*(*H*) curves were measured at different temperatures between 130 and 180 K during two cycles of increasing and decreasing magnetic field, which are shown in Fig. 6 ▸(*a*). As can be seen, at all the measurement temperatures between 160 and 180 K, the magnetization increases rapidly in the initial low-field region (below 0.1 T), which may arise from the coexistence of weakly magnetic martensite and a small amount of ferromagnetic austenite before applying the magnetic field. With further increasing magnetic field, a large magnetization jump appears at the critical field μ_0_ *H*_cr_ [as indicated in Fig. 6 ▸(*a*)], especially at temperatures between 172 and 180 K. This clearly indicates the magnetic field-induced strong meta-magnetic first-order phase transition from weakly magnetic martensite to ferromagnetic austenite. As seen from Fig. 6 ▸(*a*), at temperatures between 172 and 180 K the magnetization saturates at high magnetic fields, suggesting that the sample transforms fully into austenite under 5 T. In the temperature region of 130–166 K, only a part of the phase transition can be induced by a magnetic field of 5 T. This is because more magnetic energy and thus a higher magnetic field is needed to transform the sample fully into austenite at these temperatures, which are far below the reverse transformation temperature (Karaca *et al.*, 2009 ▸).

Comparing the *M*(*H*) curves measured during the first and second field cycles [Fig. 6 ▸(*a*)], one can see that at each temperature between 172 and 180 K the magnetization in the low-field region measured during the second increasing field is slightly higher than that measured during the first increasing field. This implies that the austenite induced by the first increasing field does not completely transform back to martensite and a small portion of the field-induced austenite remains after removal of the magnetic field in the first field cycle. On the other hand, the demagnetization curves of the first and second cycles are almost the same. Moreover, the *M*(*H*) curve in the low-field region measured during the second decreasing field coincides with that measured during the second increasing field. This implies that the residual field-induced austenite (which is only a small portion of the sample) after removal of the magnetic field in the first field cycle is no longer involved in the magnetic field-induced transition, while the reversible transition between the martensite transformed back during the first decreasing field and the austenite could occur in the second and following field cycles [similar to the case observed by Qu *et al.* (2017*b* ▸)]. Therefore, a reversible magnetic field-induced first-order phase transition between weakly magnetic monoclinic 6M martensite and ferromagnetic cubic austenite is achieved in the Ni_43.7_Cu_1.5_Co_5.1_Mn_36.7_In_13_ microwire.

**Figure 7:**
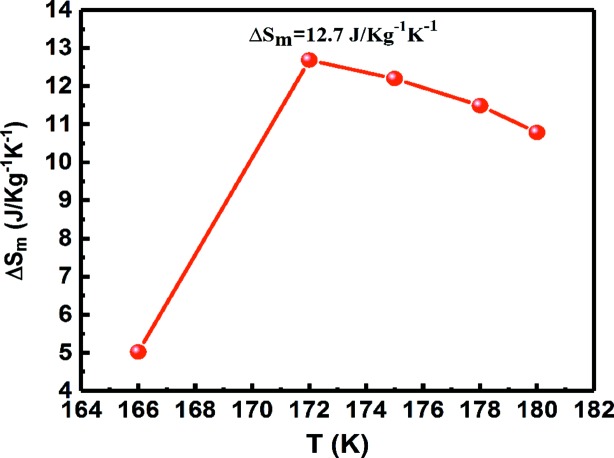
The temperature dependence of the reversible magnetic field-induced entropy change Δ*S*_m_ for a field change from 0.1 to 5 T for the Ni_43.7_Cu_1.5_Co_5.1_Mn_36.7_In_13_ microwire.

Based on the reversible magnetic field-induced first-order magnetostructural transition, a variety of magnetically driven multifunctional properties, including the magnetic shape-memory effect, magnetic superelasticity, the magnetocaloric effect, magnetoresistance and magnetothermal conductivity, could be anticipated in this microwire. As an example, we estimated the magnetocaloric effect in the Ni_43.7_Cu_1.5_Co_5.1_Mn_36.7_In_13_ microwire. The magnetic field-induced entropy change Δ*S*_m_ can be estimated from the magnetization data shown in Fig. 6 ▸(*a*). For practical applications, only the reversible Δ*S*_m_ is useful. Since the magnetic field-induced transition in the second field cycle is reversible, Δ*S*_m_ in the second field cycle is also reversible. Thus, we used the magnetization data recorded in the second field cycle to estimate the reversible Δ*S*_m_. Fig. 6 ▸(*b*) shows the temperature dependence of the critical field for the magnetic field-induced transition, μ_0_ *H*_cr_, determined from the *M*(*H*) curves recorded during the second field cycle [Fig. 6 ▸(*a*)]. The slope of the linear fit line of μ_0_ *H*_cr_ *versus T* is about −0.150 T K^−1^, which is in good agreement with the value of μ_0_Δ*H*/Δ*T*_A_ (−0.156 T K^−1^). In order to avoid overestimating Δ*S*_m_ in the case of the coexistence of a small amount of austenite and the major martensite phase in the initial state, the Clausius–Clapeyron relation was used for the correct determination of Δ*S*_m_. In the Clausius–Clapeyron relation, Δ*S*_m_ is directly related to the magnetization difference induced by the magnetic field at a given temperature (Balli *et al.*, 2009 ▸; Qu *et al.*, 2017*b* ▸; Szymczak *et al.*, 2014 ▸):
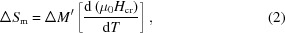
where Δ*M*′ is the difference between the magnetizations at the final field and the initial field. The magnetic field of 0.1 T is selected as the initial field because for all *M*(*H*) curves the magnetization changes rapidly below 0.1 T, which may result in numerical instabilities. The d(μ_0_ *H*_cr_)/d*T* term equals the slope of the linear fit line of μ_0_ *H*_cr_ *versus T*, which is −0.150 T K^−1^. The reversible Δ*S*_m_ for a magnetic field change from 0.1 to 5 T was estimated and is shown as a function of temperature in Fig. 7 ▸. As can be seen, Δ*S*_m_ exhibits positive values at all these temperatures, implying that the obtained magnetocaloric effect is an inverse magnetocaloric effect. The maximum reversible Δ*S*_m_ for a field change from 0.1 to 5 T is 12.7 J kg^−1^ K^−1^. Δ*S*_m_ in the present microwire exceeds that in many other magnetic microwires (Zhang *et al.*, 2016*b* ▸, 2017*b* ▸).

Our Ni_43.7_Cu_1.5_Co_5.1_Mn_36.7_In_13_ microwire shows both a pronounced magnetic field-induced magnetostructural transformation and huge tensile superelasticity as a result of a stress-induced martensitic transformation. This provides the opportunity for controlling the phase transition and optimizing the multifunctional properties under the coupling of multiple external fields (magnetic field, stress and temperature). The microwire contains neither rare earth nor toxic elements, it exhibits single-crystal-like properties but can be easily fabricated by rapid continuous wire drawing, with no post-processing required, and it has a high specific surface area. All these merits confer on this microwire great potential for applications in micro-sensors, micro-actuators, micro-magnetic refrigeration and micro-multifunctional devices used in lab-on-a-chip systems or in biomedical technology.

## 4. Conclusions   

An Ni–Cu–Co–Mn–In microwire with both a magnetic field-induced first-order magnetostructural transformation and huge tensile superelasticity has been developed. A temperature-dependent *in situ* synchrotron high-energy X-ray diffraction investigation reveals that the martensite of this Ni_43.7_Cu_1.5_Co_5.1_Mn_36.7_In_13_ microwire shows a monoclinic six-layered modulated (6M) structure (space group *P*2/*m*, No. 10) and the austenite shows a cubic structure. This microwire exhibits an oligocrystalline structure with bamboo grains, which remarkably reduces the deformation and transformation strain incompatibility, allowing the deformation and martensitic transformation to occur in a much less constrained environment. As a result, this microwire shows huge tensile superelasticity with a recoverable strain of 13%, which is in agreement with our theoretical calculations based on the crystal structure and lattice correspondence of austenite and martensite and the crystallographic orientation of the grains. This huge tensile superelasticity is in contrast with the poor tensile deformability of bulk MMSMAs.

Owing to the large magnetization difference between austenite and martensite, a pronounced magnetic field-induced magnetostructural transformation is achieved in the Ni_43.7_Cu_1.5_Co_5.1_Mn_36.7_In_13_ microwire. Based on this transformation, a variety of magnetically driven functional properties could be expected. For example, a large reversible magnetocaloric effect with a field-induced isothermal entropy change Δ*S*_m_ of 12.7 J kg^−1^ K^−1^ under 5 T is achieved in this microwire. This is the first time that a magnetic field-induced first-order meta-magnetic phase transition and excellent tensile superelasticity have been achieved simultaneously in a single material. This study may lay a foundation for exploiting the multifunctional properties under the coupling of magnetic field and stress in microwires for applications in miniature multifunctional devices.

## Supplementary Material

Additional experimental figure. DOI: 10.1107/S2052252519009102/fc5034sup1.pdf

## Figures and Tables

**Table 1:** Phase transition temperatures for the Ni_43.7_Cu_1.5_Co_5.1_Mn_36.7_In_13_ microwire measured by DSC under zero field and by PPMS under 0.05 and 5 T

	Transition temperatures (K)						
μ_0_Δ*H* (T)	*M*_s_	*M*_f_	*A*_s_	*A*_f_	*T*_M_	*T*_A_	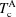
0	185.3	175.1	193.3	202.5	182.3	199.0	300.8
0.05	181.2	178.3	195.9	201.3	180.1	200.4	300.7
5	145.8	138.7	163.6	171.5	143.0	168.6	
