# Supplementary material for: Magnetic field-induced magnetostructural transition and huge tensile superelasticity in an oligocrystalline Ni–Cu–Co–Mn–In microwire
Source: IUCrJ. 2019 Jul 11;6(Pt 5):843–53. doi: 10.1107/S2052252519009102 (PMC6760440; doi:10.1107/S2052252519009102)
Supplement: Supplementary file 1 [file m-06-00843-sup1.pdf]

IUCrJ

Volume 6 (2019)

Supporting information for article:

Magnetic field-induced magnetostructural transition and huge tensile superelasticity in an oligocrystalline Ni–Cu–Co–Mn–In microwire

Zhen Chen, Daoyong Cong, Xiaoming Sun, Yin Zhang, Haile Yan, Shaohui Li, Runguang Li, Zhihua Nie, Yang Ren and Yandong Wang

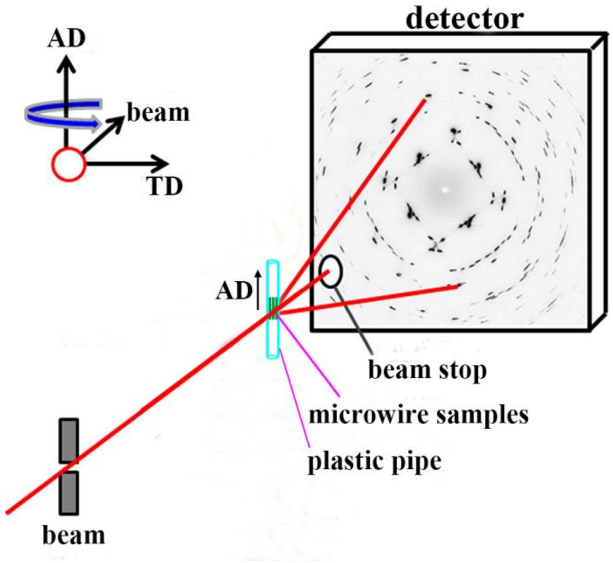

Figure. S1. Schematic illustration of the setup for synchrotron high-energy X-ray diffraction experiments. The relative orientation of the incident X-ray beam with respect to the microwires is shown. AD denotes the axial direction of the microwires and TD represents the transverse direction. Three microwires were stacked together and used for the experiments; they were rotated at a high speed during data collection.
